# Teacher Perceptions of a School-Based Support Program for Children with Cancer

**DOI:** 10.5334/cie.140

**Published:** 2024-11-20

**Authors:** Emily D. Irwin, Rachael Jewell, Jessika C. Boles, Tisha Coggin Clay

**Affiliations:** 1 Vanderbilt University Peabody College of Education Department of Psychology and Human Development, US; 2 Vanderbilt University, US; 3 Monroe Carell Jr. Children’s Hospital at Vanderbilt, US

**Keywords:** childhood illness, school re-entry, teachers, childhood cancer, pediatrics, school-based program

## Abstract

Innovations in medicine have allowed children with cancer to attend school more frequently by increasing survival rates and improving access to outpatient therapies. Children with cancer still miss a significant proportion of school attendance and participation during treatment, thereby disrupting their educational experiences. “Monkey in My Chair” is a program in the United States that connects ill children with their schoolmates during illness-related absences to support their social relationships and eventual school re-entry into the school environment. However, little is known about how this program is perceived and experienced by participating schoolteachers. Therefore, the purpose of this study was to understand teacher perceptions of the Monkey in My Chair program. One hundred and one teachers who participated in the program between 2012 and 2022 completed an electronic survey about their experiences. Overall, participants reported satisfaction with the program and indicated they would recommend it to other teachers. Most preferred and utilized components were the stuffed animal monkey and the perceived sense of connection it created among students. Participants suggested several areas in which the program can be improved, such as requesting more digital program components, expanding beyond the scope of oncology diagnoses, creating more developmentally appropriate materials, and including an instructional video. Future research is needed to understand all stakeholder experiences, including those of children with cancer and their classmates, to continue to evaluate and improve the Monkey in My Chair program.

## Teacher Perceptions of a School-Based Re-entry Support Program for Children with Cancer

An estimated more than 15,000 children ages birth to 19 are diagnosed with cancer each year in the United States. (National Cancer Institute, 2023; Siegel et al., 2023). Recent innovations in cancer treatment have increased overall five-year survival rates to around 85%, with some specific diagnoses approaching 90% survival in the United States (National Cancer Institute, 2023; Siegel et al., 2023). However, these increased survival rates often come with extended treatment plans, causing children in active cancer treatment to exhibit rates of school absenteeism more than double those of children with other chronic illnesses (French et al., 2013).

As children with cancer miss opportunities to engage in educational coursework during treatment, they also miss out on crucial social interactions. One study conducted by Boles and Winsor (2019) found children with cancer primarily associated school with social interactions. School sense of belonging, the idea that when students feel included and have a place at their school, has been shown in the literature to be essential for children with medical conditions to their psychosocial well-being and academic success (Tomberi and Ciucci, 2021). Several studies have found that re-entering school after the conclusion of treatment is also fraught with difficulty as students struggle particularly with social reintegration or ostracism suggesting these students may be feeling low levels of school sense of belonging (Arpaci and Altay, 2024; Martinez-Santos et al., 2021; Sawyer et al., 2023; Tremolada et al., 2020). A recent systematic review on the educational needs of children with cancer found that a lack of communication between schools, families, and healthcare professionals also made school re-entry more complex, especially when schools had a poor understanding of the child’s condition and needs, thereby contributing to student feelings of exclusion. (Martinez-Santos et al., 2021). While limited research exists to understand teachers’ perceptions of frequently absent students with chronic illnesses, Hen (2022) found that teachers and parents perceived school re-entry for children with cancer to be smoother when relationships with classmates and school personnel were well maintained. These findings are supported by the development of the key educational factors that have been identified in the EU to support children with chronic illnesses throughout their treatment journey and when they are returning to the classroom (Capurso and Dennis, 2017). A few of these factors include supporting relationships, making sense and constructing knowledge, and inter-institutional communication (ex. Hospital and school) (Capurso and Dennis, 2017).

Data shows that childhood cancer patients and survivors report higher rates of self-reporting bullying and social isolation both during treatment and upon school re-entry after an extended illness or hospitalization (Collins et al., 2019; Sawyer et al., 2023; Tremolada et al., 2020). Some school-based programs exist to support students while they are absent such as Panda in My Seat (A program adapted from Monkey in My Chair based in the United Kingdom) and Bear in the Chair (LeHo Project, N.D; Children Health Foundation, 2019). However, none have been empirically evaluated to understand teachers’/facilitators’ experiences of program implementation and provision (Schilling and Getch, 2018). Instead, much of the research surrounding school re-entry supports involves “do-it-yourself” style interventions created by individual schools or families.

As of yet, few studies have been identified to empirically evaluate any organized intervention that supports the school re-entry process for children with cancer (Helms et al., 2016). The rapidly changing landscape of childhood cancer care and survivorship also quickly renders existing evaluations outdated or obsolete; some have suggested that school-based support programs may reduce depression in children with cancer while also improving academic performance (Helms et al., 2016; Varni et al., 1993). Additionally, these programs have the opportunity to increase school sense of belonging, which may be a protective factor for children with medical conditions (Tomberli and Ciucci, 2021).

Moreover, those programs that included a peer education component yielded more positive peer attitudes and classmates being less fearful of the child with cancer (Benner and Marlow, 1991; DeLong, 1999; Helms et al., 2016; Katz et al., 1992; Soejima et al., 2015; Treiber et al., 1986; Varni et al., 1993). Similarly, a systematic review analyzing childhood cancer patients’ experiences with school found that families who participated in any school support program reported higher levels of school attendance and lower levels of child behavior problems than those who did not (Vance and Eiser, 2002). Lastly, programs that primarily use technology to promote school sense of belonging for children with medical conditions also show promise at increasing connectivity between students and their school (Tomberli and Ciucci, 2021).

Overall, studies have shown that when children are provided with developmentally appropriate education regarding childhood cancer, they are more likely to perceive their peers with cancer in a positive way (Nash and Weinberger, 2021; Vance and Eiser, 2002). Without this education, students may hold misconceptions about the nature of and restrictions associated with their classmate’s illness that can impede social interactions and relationships (Nash and Weinberger, 2021; Sarikaya Karabudak et al., 2019). Previous literature describes teachers feeling unprepared to support students returning to school after a long-term absence (Hen, 2022), suggesting a need for more substantial support that may be provided through programs such as Monkey in My Chair. Much of the research on the needs of teachers supporting students with chronic illness surrounds hospital schoolteachers and does not address the student’s school outside of the hospital, for example, Benigno and Fante (2020), who evaluated the stress and gratification of hospital school teachers in Italy. Further, policy fails to protect these students’ psychological well-being as much focuses on their educational needs such as Section 504 (Office for Civil Rights, 2023). Lastly, some of the key educational factors that have been identified for children with chronic illness can be addressed through a school-based support program, which could reduce the burden on families and teachers (Capurso and Dennis, 2017). The present study aims to understand teachers’ experiences participating in Monkey in My Chair, a school-based support program for students who are frequently absent due to a cancer diagnosis, and their healthy classmates, and provide future directions to support this population.

## The Monkey in My Chair Program

The Monkey in My Chair program was started in the early 2000s to help children with cancer maintain a connection with their classroom while in active treatment. The program is associated with the Love, Chloe Foundation, a non-profit organization that supports the programming. Monkey in My Chair is a free program that provides hospitals and oncology programs with “monkey kits” to hospital school teachers, Certified Child Life Specialists (members of the health care team that provide developmentally appropriate psychosocial support for children and families throughout illness or injury), or other who staff can distribute to children with cancer (Monkey in My Chair, n.d.). Families who qualify can also directly request a kit. Each kit includes: 1) a large stuffed monkey intended to take the child’s place in the classroom when they are unable to attend; 2) a children’s book to help facilitate conversation about cancer treatment between the teacher and students; 3) a teacher companion guide; and 4) access to an online platform called Monkey Message that allows the student and class to share pictures and documents) (Monkey in My Chair, 2023). (see Table 1 below)

**Table 1:** Monkey in My Chair Program Components.


ITEM	TEACHER COMPANION	MONKEY MESSAGE	STUFFED ANIMAL MONKEYS	CHILDREN’S BOOK

Description	A guidebook for teachers on how to utilize the program and resources for the classroom	An online portal for students to message their absent classmate	A large stuffed animal monkey intended to take the absent students place at school and a smaller monkey for the student to keep.	A picture book to aid in the explanation of the purpose of the monkey and their absent classmate.


### The Present Study

The purpose of this descriptive mixed methods study was to qualitatively explore the experiences of teachers in the United States who participated in the Monkey in My Chair Program between 2012 and 2022 to better understand the program’s implementation and perceived impacts. By better understanding teacher perceptions and experiences, it is possible to adjust any problematic program components and enhance helpful aspects to best support students who are chronically absent due to medical treatment. These findings will also better support the teachers and school personnel as they navigate the challenges associated with having a chronically absent student.

## Method

### Participants

The contact information for the teachers in this study was provided by the founder of the Monkey in My Chair program. Participants for this study were 104 individuals from the United States who 1) were proficient in English, 2) participated in the Monkey in My Chair program between 2012 and 2022, and 3) were teachers in the United States during the time of program participation. Randomization was not needed or used given the descriptive aims of the study. Potential participants were recruited via convenience sample by an email list of registered users from 2013–2023 generated by the founder of the program. An email was sent to a total of 1,837 recipients which yielded a response rate of 5%. Emails were sent once every three weeks for a total of three times, or until a participant completed the survey. There was no incentive for participating in the survey.

The final sample included data from 101 participants (see Table 2 below). All participants self-identified as female (100%). The majority (96%) identified as female and white with only 3% identifying as female and black and 1% with a missing response. Of the 101 participants, 56.4% reported having a master’s degree, 71.3% were 41 years or older, and nearly half (47.6%) taught PreK, Kindergarten, or first grade at the time of program use.

**Table 2:** Participant Demographics.


VARIABLE	n	%

Age		

20–25	1	1

26–30	8	7.9

31–40	20	19.8

41–50	39	38.6

51+	33	32.7

Race		

white	97	96

Black	3	3

Missing	1	1

Hispanic/Latino		

Yes	4	4.2

No	92	95.8

Missing	5	5

Highest Level of Education		

Some College	1	1

Bachelor’s Degree	40	39.6

Master’s Degree	57	56.4

Other	2	2

Missing	1	1

Grade Taught		

PreK-1st	48	47.6

2^nd^–4th	17	16.8

5th–7th	33	32.7

Other	3	3

Years of Teaching Experience		

0–5	6	5.9

6–10	19	18.8

11–15	18	17.8

16–20	16	15.8

21+	39	38.6

Missing	3	3

Total	101	100


### Analysis

Survey responses were extracted from REDCap and analyzed using SPSS. Of the 104 participant records, three were excluded due to incomplete responses leaving 101 records for analysis. Basic descriptive statistics were used to analyze demographic data and Likert scale survey responses. Open-ended questions were hand-coded using an open qualitative inductive process, line-by-line, to identify categories and themes (adapted from Boles et al., 2017; Braun and Clarke, 2006; Kriukow, 2018). Thematic analysis has been used in psychology and demonstrated to be effective at identifying patterns and themes in qualitative data (Braun and Clarke, 2006) Coding was done by two trained graduate-level research assistants independently and then compiled into a master code list. Codes included single words, phrases, and impactful statements. Thematic grouping was then done by identifying repetitive or similar words and phrases. Open-ended answers were once again reviewed by the research assistants to ensure accuracy.

### Procedure

Participants were sent an email that contained a REDCap survey link once every month for three months. Upon entering the survey, they were prompted with a statement of consent and indicated their desire to move forward with the survey. Participants then completed a short survey consisting of five demographic questions, five yes or no questions, one true or false question, five Likert scales, five open-ended questions, and four multiple choice questions. Each question asked participants to acknowledge, rate, and share details about the program that they thought helpful, unhelpful, liked, or disliked. Open-ended questions offered participants to provide recommendations to improve the program, as well as share any additional information regarding their participation.

## Results

Of the included 101 participants, the majority (78.2%) indicated the stuffed monkey as their favorite component of the program, while the included children’s book was the second favorite with 12.9% of respondents. Alternatively, when asked about their least favorite component, 72.3% of teachers said “none” with the most frequent selection being Monkey Message (6.9%). Teachers who took part in the survey reported feeling overwhelmingly positive about the Monkey in My Chair program, with 98% agreeing they enjoyed the program and 95% citing they would be “very likely” to recommend the program to another teacher with a student undergoing cancer treatment (see Table 3).

**Table 3:** Likert Scale Responses.


1–10	MEAN	STANDARD DEVIATION

How would you rank the overall program use and information?	83.88	13.43

How likely are you to recommend the Monkey in My Chair Program?	88.04	16.1

Helpfulness		

How would you describe the teacher’s companion?	64.63	22.89

How would you describe the information in the teacher’s companion?	67.82	21.52

How would you describe the included children’s book?	80.89	20.33


Additionally, line-by-line thematic coding of participants’ open-ended question responses generated three themes: 1) teacher perceptions of program components, 2) perceived program impacts, and 3) recommendations to enhance programming.

### Teacher Perceptions of Program Components

**Figure 1:**
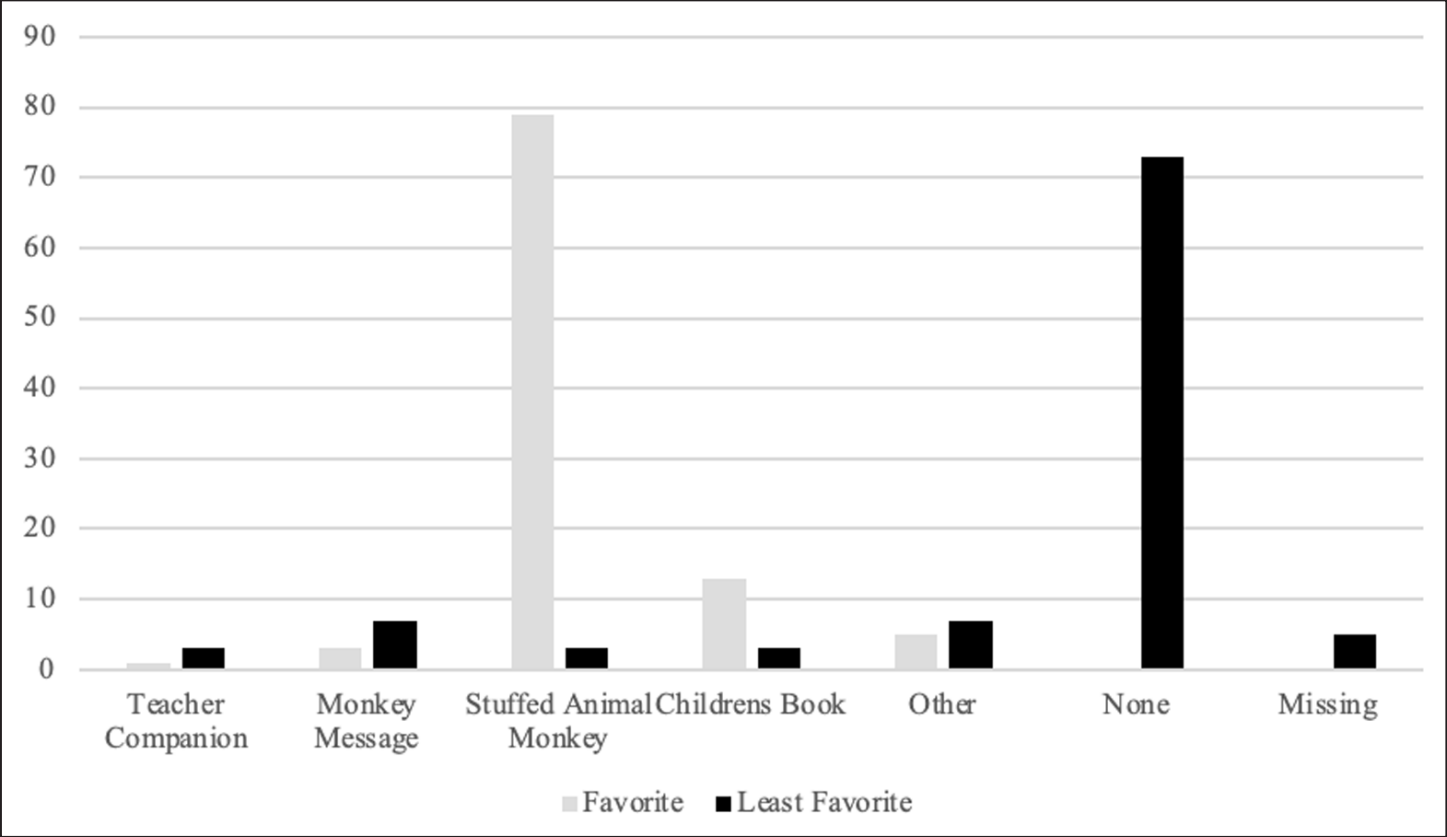
Participant Rating of Program Components. In Figure 1, teachers rated each program component.

Teachers who participated in the program largely identified the stuffed monkey as their favorite program component, with the included children’s book as a second favorite component (see Figure 1 below). However, one participant noted that “at times, it was inconvenient to bring the monkey around on campus…” Another participant stated, “Unfortunately, we did not have time to get into all the aspects of the program.”

Alternatively, some teachers chose to mention aspects of the program that they adapted themselves as their favorite part of participating. Specifically, four participants mentioned taking photos of the monkey and sending them to their absent students. One teacher said their favorite part was, “The photos we took of the monkey doing different things in our class and sending them to our student who was at (hospital name).” Lastly, three teachers noted that they did not have a favorite part and that all parts of this program were very helpful despite the challenge that comes with integrating the program.

### Perceived Impacts

Participants first described the emotional impact of the Monkey in My Chair program and the creativity they employed to individualize the program to support the needs of their absent students. When asked to enter any additional comments at the end of the survey, teachers voiced sincere praise for the program through this question. Specifically, connectedness was noted as a recurrent perceived impact, with participants reporting that the program brought their classroom and absent students together during a difficult time. One participant mentioned that “it really made a positive change for my student, who was going through a really difficult time. It brought our classroom together and made everyone feel like they were helping this student.”. Another stated, “It really helped not only my student that was absent by helping him feel connected to his class, but it helped the other students have a better understanding and empathy for others that I know they will carry with them forever.” One shared how the program offered her classroom space to talk about their absent student, “It did give us a chance to talk about our friend that was unable to be with us.”

Additionally, teachers whose students died from their cancer diagnosis or treatment mentioned the program’s unique impact in this context. As one shared, “Our monkey was there through all of it. For the kids and for our student. The monkey has a home in our school forever, to remind us of our sweet [student’s name] who lost her battle with cancer.” One teacher even mentioned, “Unfortunately, my student passed away that year, but his mom wanted me to keep his monkey. When I spoke at his funeral, I held his monkey, and even now his monkey is still in my room 8 years later.”

### Recommended Enhancements

Participants provided several recommendations for enhancing the Monkey in My Chair program or future similar programs (See Table 4). First and foremost, they expressed a strong preference for more digital program components. Specifically, many suggested the creation of “an instructional video” for teachers to accompany the program and another video for students. Additionally, three participants mentioned the desire for a social media connectivity component, and six recommended a digital platform that was “user-friendly.” Another recommendation was to include other diagnoses besides cancer, with one teacher stating they made-shifted a similar program for another student in her classroom with Crohn’s disease. Finally, teachers requested catering program components for different age groups. One participant said, “It was necessary to paraphrase to scaffold [for] my students. Reading comprehension, attention, and content differ for early childhood, middle childhood, and adolescence.”

**Table 4:** Participant Recommendations for Program Improvement.


THEME	RECOMMENDATION			

Need for Instructions	“I feel as though I was given a lot of materials for the program but was not told exactly how to utilize it.”	“A short video to show the class with an explanation of why sometimes students have to miss school and why we have the monkey”.	“Reach out to the teacher to inform her/him what the money program is about.”	“Maybe a quick introduction video with some kids who have used the program? Like 3–5 min long?”

Digital Enhancement	“Create an online component where kids can connect”.	“Utilize social media more!”	“I wish there was an online portal that my students could have sent messages directly to our absent student. If there was a component like that, I was not aware of”.	“Make the messaging component more streamlined”.

Developmentally Appropriate Materials	“Have a book that would be appropriate for older grade levels instead of just primary grades”.	“A higher-level middle school grades appropriate book”	“A book geared toward early childhood would be great”.	“It could have an option for materials to be more relatable to upper elementary students”.

Expand Diagnoses	“We have other children who have had long periods of time away from class. I think it great for cancer, but other disease and causes could be great too!”	“I used it for a child with Crohn’s disease, a child with SMA, and child with MLS who all needed to be in and out of school”.	“I would have liked an option for it to be any type of prolonged absence. But it was easy to adjust to that!”	


## Discussion

The findings of this study suggest the utility and acceptability of creating and enhancing school-based support programs for children who are chronically absent due to medical treatment in the United States. Prior research has shown that school-aged childhood cancer survivors are less engaged in peer interactions compared to their peers (Katz et al., 2011), and that school re-entry is challenging for the student with cancer, their classmates, and their teacher after treatment (Arpaci and Altay, 2024; Hen, 2022; Martinez-Santos et al., 2021; Tremolada et al., 2020). These students must remain connected to their school to have a positive school sense of belonging to protect their well-being and academic success (Tomberli and Ciucci, 2021). Factors to consider when crafting programming also include the key educational factors constructed in the EU to aid in the continuity of children with chronic illnesses’ education and school re-entry such as inter-institutional communication and facilitating relationships (Capurso and Dennis, 2017). Previously evaluated interventions have been seen to maintain connectivity and provide peer education during long-term absences (Helms et al., 2016; Katz et al., 1992), but little was yet known about what appears to be the most used program in the United States, Monkey in My Chair. Participants in this study indicated overwhelmingly positive feelings about the program and perceived an array of benefits and opportunities for enhancing this program.

The results from this study importantly suggest that teachers are not only willing to implement programming for chronically absent students, but they also perceive it to be supportive and effective for the entire classroom. Past research has shown that teachers do not feel adequately prepared to support children with medical conditions returning to school and these findings demonstrate that this program or similar programs may help aid in supporting teachers through their student’s absence (Hen, 2022). Additionally, these findings suggest the efficacy and potential transferability of implementation with other diagnoses besides cancer, as gathered from participant recommendations. Understanding that a program such as Monkey in My Chair is well-received by teachers offers further support and rationale for school-based support interventions for chronically absent students.

## Conclusion

This study was designed to understand teachers’ perceptions of a school support program for children with cancer in the United States. Although there were a few recommendations from teachers who participated in this program, such as improving the use of digital materials, creating programs for other diagnoses, and different program components for each age group, the use of the Monkey in My Chair program was perceived to be easy to use and contained helpful components for teachers. This was demonstrated through both qualitative and quantitative findings. Many teachers found that this program fostered a connection with the absent student, as well as provided peers with context for the student’s absence citing things like “a positive change” and providing a “better understanding” for the other students.

Regarding the recommendations from teachers to improve this program, many teachers thought that this program needed improved digital features. A few of the suggestions they gave were to have an instructional video for both them and their students, which does align with the lower scores for the perceived helpfulness of the “teacher companion”, a physical guidebook. Technology is now more accessible than ever and with the help of subject matter experts, video support or interactive modules may significantly enhance programming. Also, platforms such as YouTube are free, and videos can be uploaded to the platform and accessed by all. Teachers also suggested that this program be adapted for children with other chronic illnesses. Including these program enhancements in the Monkey in My Chair program would seemingly make the program more accessible and easier to implement. Minimal changes would need to be made to include other illnesses in programming except for educational materials on specific diagnoses and funding expansion.

Although some hospital schoolteachers and other healthcare personnel have anecdotally shared concerns about the inclusivity of the program, citing racial and cultural stereotypes that monkeys can be associated with, participants in this study did not call attention to this issue. However, this incidental finding may be attributable to the lack of racial and ethnic diversity in the participant sample. Future research is needed to expand upon the findings, better understand the absent child’s experiences with school-based support programming and elicit more diverse perspectives on the program.

### Limits of the Study

This study was not without limitations. First, due to the design, it is possible that survey bias such as non-response and response bias likely caused the overwhelmingly positive results. The lack of prior research on the topic and comparable programs may have influenced teachers’ perceptions and responses. Monkey in My Chair is one of the very few programs to support students who are chronically absent due to medical treatment. Therefore, any programming offered may be considered supportive when no alternative option exists. Furthermore, the participants from this study were recruited from all over the United States, from a variety of schools, both public and private. This is important to note as some teachers may have had higher levels of support from school administrators to take time and use all program components, while others may have to comply with strict curriculum guidelines. Additionally, the email addresses utilized for participant recruitment date back as far as 2013, which could imply some may not be in use anymore contributing to the low response rate. Lastly, the lack of diversity of participants may have contributed to the nature of the results. The sample size was highly heterogeneous, including white, female, and teachers over the age of 40.

### Implications for Further Research

The present study offers insights into the use of the Monkey in My Chair program which may be transferable to other school-based interventions for students with cancer and their classmates. Future research should further evaluate programming longitudinally from the child participant’s perspectives and include stakeholders such as hospital staff, school administrators, and parents/caregivers to strengthen program elements and outcomes. These studies may provide more robust data including understanding the impact on school sense of belonging and post-program outcomes. Additionally, future research should aim to diversify methodology to include more qualitative measures such as focus groups, interviewing, and observation of implementation which may help refine components and provide a unique perspective. Further recommendations include the need and further investigation of policies for schools to incorporate that require including absent students in class activities as this program or similar programming is not universally accessible. Such policy may be enacted at the district, state, or even federal level that focuses on the psychosocial needs of children with medical conditions as they navigate their illness. While a policy such as Section 504 exists to ensure the continuity and access to education for children with disabilities, including medical needs, a similar policy should be enacted to ensure the psychological well-being of these students. Lastly, future research should seek and identify local funding opportunities for programs to increase access to school support programming for patients with various diagnoses such as through government, private donors, grants, or individual schools.

## References

[1] Arpaci, T., & Altay, N. (2024). Qualitative analysis of school re-entry experiences of Turkish survivors of childhood and adolescent cancer: Parental perspective. Seminars in Oncology Nursing, 40(2), 151613. 10.1016/j.soncn.2024.151613 38413308

[2] Benigno, V., & Fante, C. (2020). Hospital School Teachers’ Sense of Stress and Gratification: An Investigation of the Italian Context. Continuity in Education, 1(1), 37–47. 10.5334/cie.14 38774532 PMC11104412

[3] Benner, A. E., & Marlow, L. S. (1991). The effect of a workshop on childhood cancer on students’ knowledge, concerns, and desire to interact with a classmate with cancer. Children’s Health Care, 20(2), 101–107. 10.1207/s15326888chc2002_5

[4] Boles, J., & Winsor, D. L. (2019). “My school is where my friends are”: Interpreting the drawings of children with cancer. Journal of Research in Childhood Education, 33(2), 225–241. 10.1080/02568543.2019.1577771

[5] Boles, J. C., Winsor, D. L., Mandrell, B., Gattuso, J., West, N., Leigh, L., & Grissom, S. M. (2017). Student/patient: The school perceptions of children with cancer. Educational Studies, 43(5), 549–566. 10.1080/03055698.2017.1312288

[6] Braun, V., & Clarke, V. (2006). Using thematic analysis in psychology. Qualitative Research in Psychology, 3(2), 77–101. 10.1191/1478088706qp063oa

[7] Capurso, M., & Dennis, J. L. (2017). Key educational factors in the education of students with a medical condition. Support for Learning, 32(2), 158–179. 10.1111/1467-9604.12156

[8] Children’s Health Foundation. (2019). Bear in the chair. Chfkids. https://www.chfkids.com/bearinthechair

[9] Collins, D. E., Ellis, S. J., Janin, M. M., Wakefield, C. E., Bussey, K., Cohn, R. J., Lah, S., & Fardell, J. E. (2019). A systematic review summarizing the state of evidence on bullying in childhood cancer patients/survivors. Journal of Pediatric Oncology Nursing, 36(1), 55–68. 10.1177/1043454218810136 30406714

[10] DeLong, M. D. (1999). Peers’ knowledge and attitudes toward a classmate with cancer: An evaluation of a school reintegration program. ProQuest Dissertations Publishing.

[11] French, A. E., Tsangaris, E., Barrera, M., Guger, S., Brown, R., Urbach, S., Stephens, D., & Nathan, P. C. (2013). School attendance in childhood cancer survivors and their siblings. The Journal of Pediatrics, 162(1), 160–165. 10.1016/j.jpeds.2012.06.066 22835883

[12] Helms, A. S., Schmiegelow, K., Brok, J., Johansen, C., Thorsteinsson, T., Simovska, V., & Larsen, H. B. (2016). Facilitation of school re-entry and peer acceptance of children with cancer: a review and meta-analysis of intervention studies. European Journal of Cancer Care, 25(1), 170–179. 10.1111/ecc.12230 25204197

[13] Hen, M. (2022). Mothers’ and teachers’ experience of school re-entry after a child’s prolonged absence due to severe illness. Psychology in the Schools, 59(6), 1122–1134. 10.1002/pits.22666

[14] Katz, E. R., Varm, J. W., Rubenstein, C. L., Blew, A., & Hubert, N. (1992). Teacher, parent, and child evaluative ratings of a school reintegration intervention for children with newly diagnosed cancer. Children’s Health Care, 21(2), 69–75. 10.1207/s15326888chc2102_1 10117965

[15] Katz, L. F., Leary, A., Breiger, D., & Friedman, D. (2011). Pediatric cancer and the quality of children’s dyadic peer interactions. Journal of Pediatric Psychology, 36(2), 237–247. 10.1093/jpepsy/jsq050 20522423 PMC3107586

[16] Kriukow, J. (Academic). (2018). Coding: line-by-line coding [Video]. Sage Research Methods. 10.4135/9781529623413

[17] Leho Project. (n.d). Panda in my seat! Learning at home and in the hospital. https://www.lehoproject.eu/en/toolkit/97-there-s-a-panda-in-my-seat

[18] Martinez-Santos, A. E., Fernandez-De-La-Iglesia, J. D. C., Sheaf, G., & Coyne, I. (2021). A systematic review of the educational experiences and needs of children with cancer returning to school. Journal of Advanced Nursing, 77(7), 2971–2994. 10.1111/jan.14784 33598984

[19] Monkey in My Chair. (n.d.). About the program. Monkey in My Chair. https://www.monkeyinmychair.org/about-the-program/

[20] Monkey in My Chair. (2023). Monkey in my Chair. http://www.monkeyinmychair.org

[21] Nash, J. G., & Weinberger, N. (2021). You’re brave, i’ll be your friend: Children’s evaluations of peers with cancer. Psychology in the Schools, 58(6), 1114–1132. 10.1002/pits.22492

[22] National Cancer Institute. (2023). “Cancer in children and adolescents.” https://www.cancer.gov/types/childhood-cancers/child-adolescent-cancers-fact-sheet

[23] Office for Civil Rights. (2023). Protecting students with disabilities. U.S. Department of Education. https://www2.ed.gov/about/offices/list/ocr/504faq.html

[24] Sarikaya Karabudak, S., Çalışır, H., & Öner, H. (2019). Healthy Children’s knowledge and perception on cancer. Child Indicators Research, 13(1), 279–299. 10.1007/s12187-019-09686-8

[25] Sawyer, J. L., Mishna, F., Bouffet, E., Saini, M., & Zlotnik-Shaul, R. (2023). Bridging the gap: Exploring the impact of hospital isolation on peer relationships among children and adolescents with a malignant brain tumor. Child & adolescent social work journal: C & A, 40(1), 91–105. 10.1007/s10560-021-00764-x 34025015 PMC8130807

[26] Schilling, E. J., & Getch, Y. Q. (2018). School reentry services for students with chronic health conditions: An examination of regional practices. Psychology in the Schools, 55(9), 1027–1040. 10.1002/pits.22154

[27] Siegel, R. L., Miller, K. D., Wagle, N. S., & Jemal, A. (2023). Cancer statistics, 2023. CA: A Cancer Journal for Clinicians, 73(1), 17–48. 10.3322/caac.21763 36633525

[28] Soejima, T., Sato, I., Takita, J., Koh, K., Maeda, M., Ida, K., & Kamibeppu, K. (2015). Support for school reentry and relationships between children with cancer, peers, and teachers. Pediatrics International, 57(6), 1101–1107. 10.1111/ped.12730 26083836

[29] Tomberli, L., & Ciucci, E. (2021). Sense of school belonging and paediatric illness: A scoping review. Continuity in Education, 1, 121–134. 10.5334/cie.32 PMC11104300 38774888

[30] Treiber, F., Schramm, L., & Mabe, P. (1986). Childrens knowledge and concerns towards a peer with cancer – a workshop intervention approach. Child Psychiatry and Human Development, 16(4), 249–260. 10.1007/BF00706481 3743177

[31] Tremolada, M., Taverna, L., Bonichini, S., Pillon, M., Biffi, A., & Putti, M. C. (2020). Pediatric patients treated for leukemia back to school: A mixed-method analysis of narratives about daily life and illness experience. Behavioral Sciences, 10(7), 107. 10.3390/bs10070107 32630265 PMC7407376

[32] Vance, Y. H., & Eiser, C. (2002). The school experience of the child with cancer. Child: Care, Health & Development, 28(1), 5–19. 10.1046/j.1365-2214.2002.00227.x 11856182

[33] Varni, J. W., Katz, E. R., Colegrove, R., & Dolgin, M. (1993). The impact of social skills training on the adjustment of children with newly diagnosed cancer. Journal of Pediatric Psychology, 18(6), 751–767. 10.1093/jpepsy/18.6.751 8138868

